# Fully Inkjet-Printed Biosensors Fabricated with a Highly Stable Ink Based on Carbon Nanotubes and Enzyme-Functionalized Nanoparticles

**DOI:** 10.3390/nano11071645

**Published:** 2021-06-23

**Authors:** Mijal Mass, Lionel S. Veiga, Octavio Garate, Gloria Longinotti, Ana Moya, Eloi Ramón, Rosa Villa, Gabriel Ybarra, Gemma Gabriel

**Affiliations:** 1 INTI-Micro y Nanotecnologías, Instituto Nacional de Tecnología Industrial (INTI), San Martín, Buenos Aires B1650WAB, Argentina; mmass@inti.gob.ar (M.M.); lveiga@inti.gob.ar (L.S.V.); ogarate@inti.gob.ar (O.G.); glonginotti@inti.gob.ar (G.L.); 2 Institut de Microelectrònica de Barcelona, IMB-CNM (CSIC), Campus Universitat Autònoma de Barcelona, Cerdanyola del Vallès, 08193 Barcelona, Spain; ana.moya@eurecat.org (A.M.); eloi.ramon@imb-cnm.csic.es (E.R.); rosa.villa@imb-cnm.csic.es (R.V.); 3 CIBER de Bioingeniería, Biomateriales y Nanomedicina (CIBER-BBN), 28029 Madrid, Spain

**Keywords:** biosensors, electrochemical detection, inkjet printing, carbon nanotubes, carbon-ink electrodes, silica nanoparticles

## Abstract

Enzyme inks can be inkjet printed to fabricate enzymatic biosensors. However, inks containing enzymes present a low shelf life because enzymes in suspension rapidly lose their catalytic activity. Other major problems of printing these inks are the non-specific adsorption of enzymes onto the chamber walls and stability loss during printing as a result of thermal and/or mechanical stress. It is well known that the catalytic activity can be preserved for significantly longer periods of time and to harsher operational conditions when enzymes are immobilized onto adequate surfaces. Therefore, in this work, horseradish peroxidase was covalently immobilized onto silica nanoparticles. Then, the nanoparticles were mixed into an aqueous ink containing single walled carbon nanotubes. Electrodes printed with this specially formulated ink were characterized, and enzyme electrodes were printed. To test the performance of the enzyme electrodes, a complete amperometric hydrogen peroxide biosensor was fabricated by inkjet printing. The electrochemical response of the printed electrodes was evaluated by cyclic voltammetry in solutions containing redox species, such as hexacyanoferrate (III/II) ions or hydroquinone. The response of the enzyme electrodes was studied for the amperometric determination of hydrogen peroxide. Three months after the ink preparation, the printed enzyme electrodes were found to still exhibit similar sensitivity, demonstrating that catalytic activity is preserved in the proposed ink. Thus, enzyme electrodes can be successfully printed employing highly stable formulation using nanoparticles as carriers.

## 1. Introduction

Printed electronics are expected to have a great impact on the manufacturing of functional electronic devices [1,2,3,4,5,6,7], especially in the biomedical field, where the fabrication of low-cost single-use sensors and biosensors have led to interesting applications, such as personalized medicine [8,9,10,11,12]. Inkjet printing (IJP) is one of the most used printing technologies for sensor production because it allows low production costs and high mass-production of devices with the desired reproducibility [13,14,15]. A variety of functional materials can be digitally deposited by IJP in microscale dimensions at low temperatures on a wide variety of substrates. Furthermore, as a non-contact, mask-less deposition approach, it reduces fabrication time and costs, and permits customized geometries [16,17,18,19]. Recent research has also demonstrated that inkjet printing is a reliable solution for the fabrication of sensors and biosensors [20,21,22,23], making the technology advantageous for the development of green electronics [24] and reliable, easy-to-use diagnostic tests.

Specifically in the biosensing field, IJP benefits from its versatility and high resolution for the development of prototypes. However, IJP of biomolecules presents some challenges, especially regarding molecular stability. In this respect, non-specific adsorption of biomolecules onto the ink chamber walls and maintaining stability during printing, where the molecules suffer thermal and/or mechanical stress, have been reported as two major issues for IJP of biomolecules [21,25].

A range of additives have been employed in order to maintain the stability of biomolecules [26] with varying results. Although it is expected that some enzymes immobilized on a surface might exhibit a lower catalytic activity, it is well known that surface-immobilization often leads to improved stability, since their aggregation is no longer possible. Furthermore, as indicated by Rodrigues et al. [27], enzyme rigidification may lead to preservation of the enzyme properties under drastic conditions in which the enzyme tends to become distorted, thus decreasing its activity. Finally, as has been reported by Hoarau et al., methods of enzyme attachment can be fine-tuned so that activity and stability can be greatly enhanced [28].

On the other hand, carbon-based nanomaterials, especially carbon nanotubes (CNTs) and graphene, have been recognized as convenient materials for the construction of biosensors, since they can be employed as solid supports for immobilization of biomolecules and also provide a high electronic conductivity [29]. The high surface to volume ratio of CNTs enables an increase in the number of immobilized biomolecules, as well as reducing the time response of the biosensors [30]. In order to immobilize enzymes onto CNTs, they are usually oxidized to produce carboxylic groups that can act as anchoring points. For instance, a usual method to immobilize proteins onto CNTs is via the carbodiimide crosslinking reaction between carboxylic groups formed on the CNTs and free amino groups of enzymes [31]. However, it is well known that the oxidation of CNTs has a negative impact on their conductive and mechanical properties. Therefore, alternative methods of immobilization of biomolecules, which do not affect CNT electronic properties, are of interest.

The use of particulated carriers such as polystyrene microspheres, onto which biomolecules can be immobilized, has been proposed as an alternative to direct linking to CNTs for the preparation of inks for printed enzyme electrodes [32]. This approach allows enzyme immobilization without significantly affecting the electrical and electrochemical behavior of CNTs. However, inks containing microspheres are not suitable for IJP, since particles with a size higher than 200 nm cause nozzle clogging. This problem might be solved by the use of an adequate nanocarrier and, in this sense, silica nanoparticles seem a convenient material of choice. Silica nanoparticles can be prepared by the Stöber method with a low dispersity and a controlled size between 9 and 800 nm [33], amenable to IJP. Furthermore, the surface of silica particles can be easily functionalized to provide amino or carboxylic groups onto which enzymes can be covalently linked [34].

In this paper, we propose a general approach to increase the stability of biomolecules in inks and during the printing by immobilizing enzymes onto silica nanoparticles. The incorporation of these nanoparticles in a single walled carbon nanotube (SWCNT) conductive ink allows a simple and general procedure for the preparation of IJP enzyme electrodes. Our results show that this approach could be used to immobilize a considerable quantity of biomolecules without significant loss of their biological activity. As a proof a concept, we present the results for the preparation of an enzyme electrode prepared with horseradish peroxidase (HRP).

## 2. Materials and Methods

### 2.1. Synthesis of SiO_2_ Nanoparticles

Spherical SiO_2_ nanoparticles (SNPs) were prepared by a sol-gel process by controlled hydrolysis of tetraethyl orthosilicate (TEOS) in batch synthesis at room temperature. Firstly, 13.5 mL of deionized H_2_O, 24.5 mL of anhydrous EtOH and 1.22 mL of concentrated NH_3_ were placed together in a glass flask. Then 830 µL of TEOS were quickly added under vigorous magnetic stirring (800 rpm) and the flask was closed to avoid reagents evaporation. After 15 min, the solution turned into a pale blue-white solution indicating the presence of colloidal SiO_2_. The solution was left covered overnight on a rotary mixer. To remove by-products and solvents after the synthesis of the silica particles, they were centrifuged at 3000 rpm for 30 min (Mikro 1200, Hettich, Tuttlingen, Germany) and gently resuspended in 10 mL deionized water. After repeating the washing process three times, hydrodynamic size was measured by dynamic light scattering (DLS) with a DynaProNanostar from Wyatt Technology (Santa Barbara, CA, USA). Finally, the particles were dried until a solid was obtained, and then they were placed in an oven at 120 °C and under vacuum for 48 h. After this stage, they were allowed to cool and then resuspended in H_2_O. The solid was placed in a Falcon^TM^ type tube and H_2_O was added until total resuspension was achieved. Finally, they were centrifuged again at 6000 rpm for 20 min with the addition of absolute EtOH twice. All chemicals were of reagent grade and used without further purification. The size and distribution were analyzed using field emission scanning electron microscopy (Quanta 250, FEI, Waltham, MA, USA).

### 2.2. Preparation of SNP-HRP

#### 2.2.1. Functionalization of SNPs

Firstly, the dried silica nanoparticles were treated at 120 °C for 24 h to ensure the consolidation of the inorganic structure. Then, the particles were resuspended in 1.5 mL of EtOH and 200 µL of APTES was added. The reaction was allowed to stir overnight to obtain NH_2_ modified silica particles. Subsequently, they were centrifuged at 14,000 rpm for 5 min and washed with EtOH and three times with dimethylformamide (DMF). The SiO_2_-NH_2_ NPs were modified into carboxylic groups. Although several methods of covalent enzyme immobilization may be used, our experience shows that enzymes conjugated using carbodiimide present a higher activity. Thus, amino groups in the NPs were changed to carboxylic groups in order to link them to amino residues in the enzyme forming an amide covalent link. The NH_2_-modified particles dispersed in DMF were added to a previously prepared solution of 5 mL of 1% anhydrous succinic and 1.9 mL of pyridine. The mixture was stirred overnight. The nanoparticles were washed twice in DMF and resuspended in 5% HCl for 5 min. Finally, the particles were centrifuged at 8000 rpm for 10 min and resuspended in deionized water until the pH of deionized water was reached. The NPs were resuspended in 2 mL of 0.1 M phosphate buffer pH 7.0.

#### 2.2.2. HRP Immobilization onto SiO_2_ Particles

HRP was immobilized by coupling the amine groups of the enzyme with the carboxylic groups of the SNPs. Firstly, the carboxylic groups were activated by adding 500 µL of a mixture of 0.1 M EDC and 25 mM NHS (in 0.1 M phosphate buffer pH 7.0) to 500 µL of COOH-modified particles. The mixture was incubated for 60 min with low vortexing. It was washed twice with 500 µL of 0.1 M phosphate buffer solution of pH 7.0 and subsequently 80 µL of HRP (0.002 g/80 µL in phosphate buffer) was added. They were left under stirring for 2 h at room temperature. Then, the particles were centrifuged for 20 min at 3000 rpm to avoid enzyme denaturation. The supernatant was removed and gently resuspended in 500 µL of phosphate buffer. The above process was repeated 5 times. Finally, the SNPs were resuspended in 4 mL of phosphate buffer and stored in a refrigerator.

### 2.3. SWCNT Aqueous Ink

Single-walled carbon nanotubes (SWCNT) carboxylic acid functionalized with 90% carbon basis, with a diameter of 4–5 nm and a length of 0.5–1.5 µm (bundle dimensions, Sigma-Aldrich, St. Louis, MO, USA), sodium dodecyl sulfate (Sigma-Aldrich Chemie GmbH, Schnelldorf, Germany) and deionized water (conductivity less than 1 μS cm^−1^) were used for the preparation of the aqueous ink. Two inks were formulated with different concentrations of SWCNT: 7.5 mg/mL and 5 mg/mL. The first (SWCNT-7.5) was formulated with 11.2 mg of SWCNT mixed with 8 mg of sodium dodecyl sulfate (SDS), which acts as a surfactant, in an Eppendorf^®^ tube with 1.5 mL of deionized water at room temperature; while the second ink (SWCNT-5) was formulated with 7.1 mg of SWCNT mixed with 5.3 mg of SDS in 1.5 mL of deionized water. These mixtures were sonicated for 30 min and then centrifuged at 18,000 rpm for 10 min. The supernatant was used. The inks were allowed to stabilize for at least 12 h and then sonicated it again for 30 min before use. Both inks were prepared to be printed with IJP.

### 2.4. SWCNT-SNP-HPR Bio-Inks

An aqueous bio-ink was formulated by incorporating silica nanoparticles (SNP, with a mean diameter of 66 nm ± 8 nm) with covalently immobilized HRP to the SWCNT ink prepared as previously described, fulfilling the necessary rheological conditions for IJP. In order to find an adequate formulation with good electrochemical response and easy printing, several inks were prepared combining the two carbon nanotubes inks (SWCNT-7.5 and SWCNT-5) with different concentrations of the enzyme-functionalized silica nanoparticles (SNP-HRP) (Table 1).

### 2.5. IJP Electrodes Process

A three-electrode electrochemical cell was designed so that the dimensions were compatible with the electrodes used for a previously reported portable multipotentiostat [34] (Supplementary Information Figure S1). Printing patterns were made using the Electronic Design Automation (EDA) layout software and imported with the Dimatix Bitmap editor software. For the development of the conductive path, the working electrodes (WE) with a geometric surface area (GSA) of 0.78 mm^2^ and counter electrode (CE) with a GSA 5.25 mm^2^, a commercially low curing Au nanoparticle ink were used (Drycure Au-JB 1010B, C-INK Co., Okayama, Japan). For the reference electrode (RE) with a GSA 2.55 mm^2^, a silver nanoparticle ink (DGP-40LT-15C from the firm ANP, Sejong, Korea) was used. The passivation and protective layer of the electrodes was done using a dielectric PriElex^®^ SU-8 ink (MicroChem, Westborough, MA, USA). Finally, a circular area with a diameter of 1 mm was printed onto the WE with the carbon nanotubes water-based ink. All inks show drop-on-demand (DoD) inkjet compatible specifications.

The manufacturing process was performed without the need of temperature and humidity control in a standard laboratory environment. All electrodes were fabricated on a 125 µm thick polyethylene terephthalate (PET) substrate (Melinex ST504, DuPont Teijin Films, Chester, VA, USA), without any extra surface treatment, using a DoD Dimatix Materials Printer (DMP 2831 from Fujifilm Dimatix, Santa Clara, CA, USA) and three disposable and fillable cartridges containing 16 individually addressable nozzles each with a diameter of 21.5 µm and 10 pL nominal droplet volume (DMC-11610 from Dimatix Fujifilm, Lebanon, NH, USA). Each cartridge was filled with the above commented inks. Several samples of WEs were manufactured, combining different concentrations of carbon nanotubes and functionalized nanoparticles and also different numbers of layers. In the case of WEs printed with bio-ink (SWCNT-SNP-HRP), the reduction of printing times was prioritized so they were only manufactured with 12 and six layers. The details of these combinations can be seen in Table 1.

### 2.6. Fabrication of Inkjet Printed Electrode

**Figure 1:**
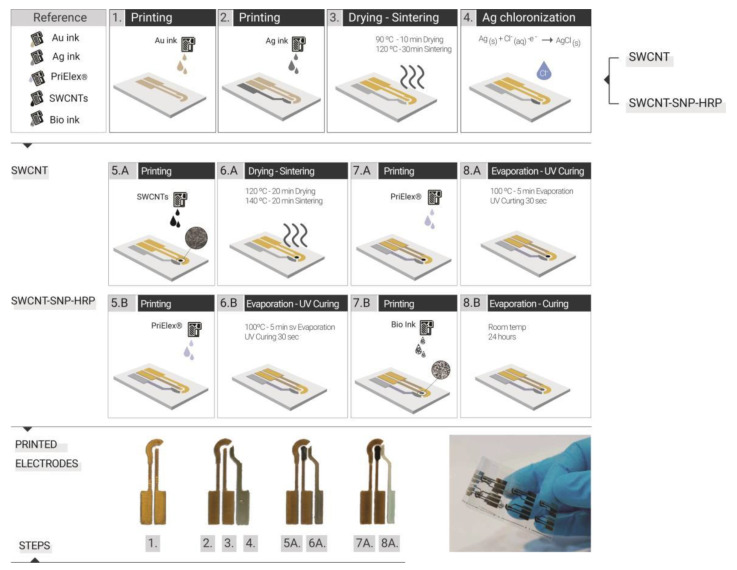
(**Top**) Fabrication steps of three-electrode electrochemical cell based on IJP. SWCNT: WE printed only with SWCNT and SWCNT-SNP-HRP: WE printed with bio-inks. (**Bottom**) Photograph of the printed electrodes steps and several all inkjet printed electrochemical sensors onto a flexible substrate.

**Figure 2:**
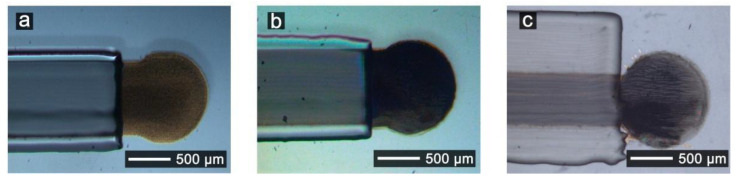
Optical microscopy images of printed electrodes: (**a**) 1-layer Au, (**b**) 12-layer SWCNT (7.5 mg/mL of SWCNT), and (**c**) 12-layer SWCNT-SNP-HRP (5 mg/mL of SWCNT and 5 mg/mL of SNP-HRP).

**Figure 3:**
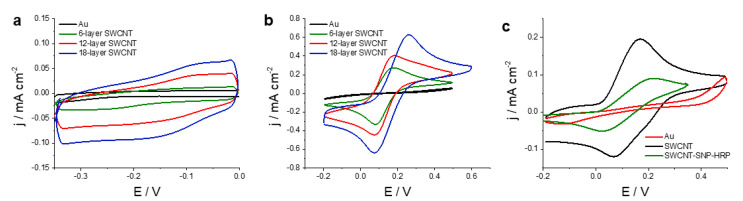
Electrochemical response of printed electrodes. (**a**) CVs in PBS buffer of pH 7.4 obtained for Au and 6-, 12-, and 18-layer SWCNT printed electrodes prepared with a concentration of 7.5 mg/mL. (**b**) CVs obtained for Au and 6-, 12-, and 18-layer SWCNT printed electrodes prepared with a concentration of 7.5 mg/m in a solution of 10 mM hexacyanoferrate (III/II) and 0.1 M KNO_3_. (**c**) CVs obtained for Au printed electrode, a 12-layer of 7.5 mg/mL concentration of SWCNT printed electrode and a 12-layer SWCNT-SNP-HRP printed electrode with 5 mg/mL and 5 mg/mL concentration of SWCNT and SNP-HRP in a solution of 4 mM hydroquinone in PBS of pH 7.4. All voltammograms were acquired at a scan rate of 0.05 V s^−1^.

**Figure 4:**
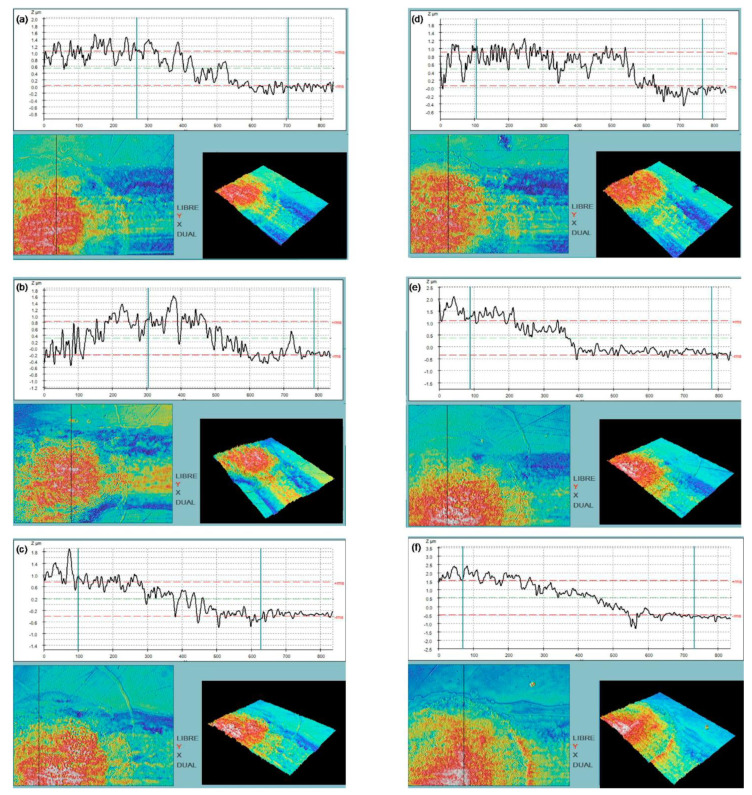
Confocal relief images and profilometry of WE printed with (**a**) 6-, (**b**) 12-, (**c**) 18-layer of SWCNT-5 ink (**left**) and (**d**) 6-, (**e**) 12-, (**f**) 18-layer of SWCNT-7.5 ink (**right**).

**Figure 5:**
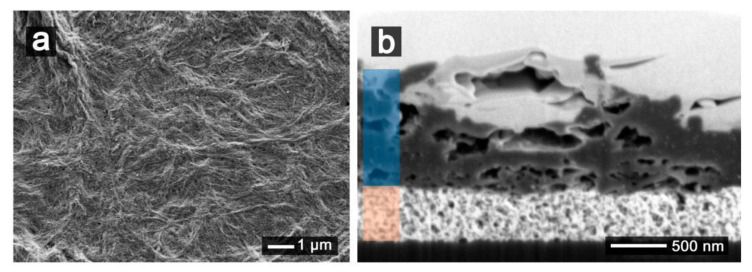
2-layer Au WE printed with 12-layer of 7.5 mg/mL of SWCNT (**a**) SEM image of the top view of its surface and (**b**) the corresponding cross section. Au layers are shaded in orange and the SWCNTs ones are shaded in blue.

**Figure 6:**
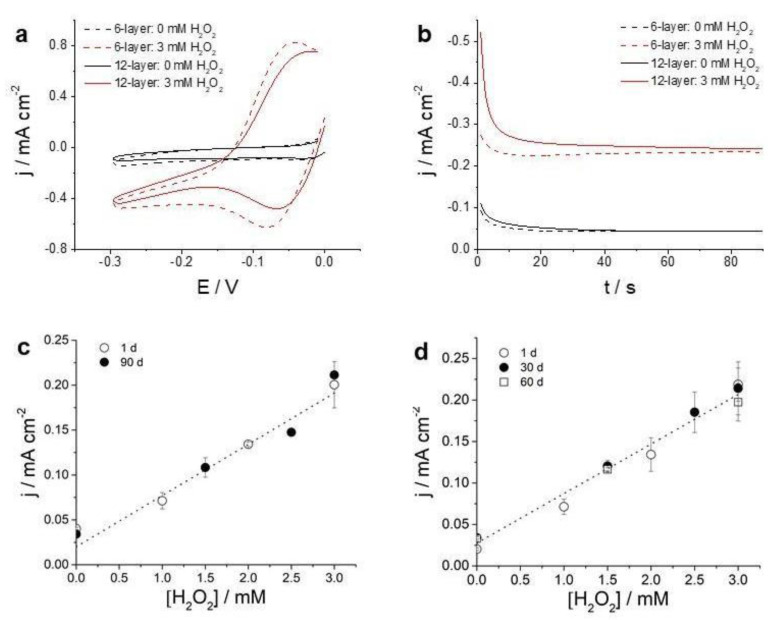
(**a**) CVs and (**b**) chronoamperometric curves for an applied potential of −0.23 V obtained for a 6-layer (dotted lines) and 12-layer (solid lines) with printed SWCNT-SNP-HRP with a concentration of 5 mg/mL of SWCNT and 5 mg/mL of SNP-HRP, respectively, without and after the addition of H_2_O_2_ to a final concentration of 3 mM in a solution of 4 mM hydroquinone in PBS of pH 7.4 at a scan rate of 0.05 V s^−1^. (**c**) Dependence of current density on H_2_O_2_ concentration for two SWCNT-SNP-HRP (6-layer with 5 mg/mL and 5 mg/mL concentration of SWCNT and SNP-HRP, respectively) electrodes printed with 1 day (open circles) and 90 days (full circles) after ink preparation. Linear regression is shown as a dotted line. Current was measured by chronoamperometry at 60 s with an applied potential of −0.23 V in 0.1 M phosphate buffer of pH 7.4 and 4 mM hydroquinone. (**d**) Dependence of current density on H_2_O_2_ concentration for three SWCNT-SNP-HRP electrodes measured 1, 30 and 60 days after being prepared under same conditions as described for Figure 6c.

**Figure 7:**
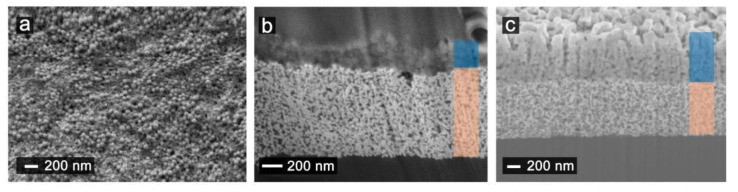
(**a**)Top view SEM images of surface of 12-layer SWCNT-SNP-HRP printed electrode. (**b**,**c**) Focused ion beam cross-section image of electrodes printed showing the different layers and SiO_2_ nanoparticles for (**b**) a 6-layer SWCNT-SNP-HRP (7.5 mg/mL of SWCNTs and 1 mg/mL of SNP-HRP) printed electrode and (**c**) of 12-layer SWCNT-SNP-HRP (5 mg/mL of SWCNTs and 5 mg/mL of SNP-HRP) printed electrode. Orange and blue shades correspond to printed Au and SWCNT-SNP-HRP films, respectively.

Optimization of the IJP process was performed as previously reported [35]. The manufacture of the electrodes was carried out following the sequence of steps as shown in Figure 1, in which SWCNT illustrates the strategy used for the printed electrodes with SWCNT ink and SWCNT-SNP-HRP the WE printed with bio-ink. This second strategy assumed that the enzymes functionalized in the bio-ink could not be exposed to temperatures higher than 37 °C, as this could affect the enzyme activity. As a first step (Figure 1-1), we printed the WE, CE and conductive paths with the Au nanoparticles ink, setting a drop spacing (DS) of 15 µm, which is equivalent to a printing resolution of 1693 dpi (dots per inch). Subsequently, the cartridge containing the Au ink was replaced with one containing Ag nanoparticle ink to print the pseudo reference electrodes (pRE) (Figure 1-2). In this case, the printing was done with a DS of 40 µm (resolution of 635 dpi). They were then subjected to thermal drying of 90 °C for 10 min and then to a sintering process in an oven 30 min at 120 °C (Figure 1-3). This step is where the final electrical properties of the inks are achieved. The pRE was chlorinated by cyclic voltammetry (CV) in 0.1 M HCl, scanning potential from 0 to 0.2 V against Ag/AgCl commercial RE (Metrohm, Herisau, Switzerland Germany) and Pt CE (Metrohm) at 20 mV/s to obtain a stable pRE (Figure 1-4) [36]. For the WE printed with the SWCNT ink, the next step was the printing of the WE area of 1 mm in diameter with the CNT water-based ink with a DS of 15 µm (1693 dpi) (Figure 1-5.A). Subsequently, they were subjected to a drying process of 20 min at 120 °C and then sintering at 140 °C for another 20 min (Figure 1-6.A). Afterwards, the PriElex^®^ SU8 dielectric ink was printed with a DS of 15 µm (1693 dpi) as a protective layer for the conductive paths and delimiting the active area for the WE and the contact pads of the electrodes (Figure 1-7.A). In this case, the curing was carried out first on a hot plate at 100 °C to evaporate the solvents and then with UV lamp exposure for 30 s, to generate the polymerization of the ink by cross-linking (Figure 1-8.A). For the electrodes printed with the bio-ink (SWCNT-SNP-HRP), after the stage of shaping the conductive paths of the electrodes, the dielectric layer was also printed with PriElex^®^ SU8, in the same way as previously described (Figure 1-5.B,6.B). In the last step, the bio-ink was printed on the active area of WE also with a DS of 15 µm (Figure 1-7.B), but in this case the curing was carried out at room temperature for 24 h (Figure 1-8.B). This curing strategy assumed that the bio-ink enzymes could not be subjected to higher than room temperature in order to avoid damage and losing enzymatic activity in the electrodes.

### 2.7. Microscopic and Electrical Characterization of Electrodes

The printed electrodes were morphologically characterized with 3D optical perfilometer, confocal mode (MS10 PLµNEOX Sensorfare, Terrassa, Barcelona, Spain) and optical microscopy (DM 4000M, Leica, Tokyo, Japan). WE diameters were measured with the ImageJ image processing program (LOCI, University of Wisconsin, Madison, WI, USA). Scanning Electron Microscopy (SEM, Auriga-40, Carl Zeiss, Jena, Germany) images were also taken of surface and the cross section made with a Focused Ion Beam (FIB, Zeiss 1560XB) of electrodes printed with the hybrid ink. SEM images were obtained from a cast drop deposition of SNP with a FEI Quanta 250 cold field SEM microscope operated at 30 kV and the mean diameter was determined as the average size of 100 nanoparticles. Resistance of a conductive layer was determined with a semiconductor parameter analyzer (B1500A, Agilent, Santa Clara, CA, USA) connected to a semi-automatic probe station (Cascade Microtech SUMMIT 12161B-6, Beaverton, OR, USA). The resistance of the conductive paths was measured with two tips of the probe station connected to two SMUs of the B1500A between both ends. A fixed voltage was injected (starting at 0.01 V with −0.0001 V steps) and the current through the path was measured.

### 2.8. Electrochemical Measurements

The electrochemical characterization of the sensors was performed with an 8-channel potentiostat 1030A Electrochemical Analyzer (CH Instruments, Bee Cave, TX, USA). Control experiments were performed using a commercial Ag/AgCl (3 M KCl) RE (DRIREF-2, World Precision Instrument, Sarasota, FL, USA) and 99.9% platinum wire (Alfa Aesar, GmbH & Co KG, Karlsruhe, Germany) as CE. All reagents were of analytical grade and used as received.

## 3. Results and Discussion

The main goal of this work was to develop an ink formulation with enzymes immobilized onto nanoparticles in order to increase the stability of the enzymes in inkjet printed electrodes. However, several issues had to be taken into account to achieve this end. The most challenging ones were to achieve good printability avoiding nozzle obstruction, a good electrochemical response towards redox probes, and an adequate analytical performance of the enzyme-based electrodes. Therefore, the choice of nanomaterials with optimum characteristics, their concentration, and the number of printed layers was carefully considered and assessed.

Figure 2 shows optical microscopy images of different WE: (a) a Au electrode, (b) a 12-layer SWCNT (7.5 mg/mL) electrode, and (c) a 12-layer SWCNT-SNP-HRP electrode (with 5 mg/mL and 5 mg/mL concentration of SWCNT and SNP-HRP, respectively). These are typical images representative of the obtained electrodes. Both Au and SWCNT printed electrodes presented a homogeneous surface in the optical microscopy images. However, in Figure 2c, printing lines can be observed on the SWCNT-SNP-HRP electrode. The characteristic gold color is seen in the Au electrode, while both SWCNT electrodes present a dark color completely covering the underlying Au surface. The SWCNT electrode in Figure 2b is darker than the SWCNT-SNP-HRP one, probably due to the different mass of SWCNT deposited (3 µg vs. 2 µg). As the number of printed layers of SWCNTs increases, the black color becomes more homogeneous and intense on the overall electrode area due to the presence of nanotubes (Supplementary Information Figure S2).

The electrodes were designed to have an area of 0.78 mm^2^ and a diameter of 1000 µm. The actual measured diameters were 1064 µm, 1126 µm and 1129 µm for Au, SWCNT and SWCNT-SNP-HRP electrodes, respectively. Therefore, the dimensions, as well as the area of the electrodes, were close to those of the original design, which confirm the compatibility of the materials and the printing process. Finally, it can be seen that the insulating layers effectively covers the conductive paths and delimits an approximate circular area.

The resistance along the Au printed paths with a length of 13.5 mm was measured and an average resistance value of 63 ± 12 Ω (standard deviation) was obtained. Therefore, negligible ohmic falls were to be expected due to the conductive paths. Typical current values were in the order or below 10 µA, so *IR* drops in the conductive paths were below 1 mV.

The SWCNTs concentration used in the inks had a major effect on the printability. When concentrations higher than 7.5 mg/mL were used, the nozzles clogged during the first impressions. Electrodes could be printed using SWCNT inks with a concentration of 7.5 mg/mL. However, after a few (from 5 to 8) days of use, the nozzles clogged and the printhead had to be discarded. In contrast, printability greatly improved when the SWCNT concentration was lower than 5 mg/mL, being possible to print for at least 3 months keeping the nozzles working. In addition, inks prepared with this SWCNT concentration were less sensitive to printing parameters such as waveform, voltages applied to the nozzle, etc., which had to be carefully chosen for more concentrated SWCNT inks (Supplementary Information Figure S3). The final mass of deposited SWCNT could be estimated using the concentration of SWCNT, drop spacing and the volume of ink deposited per layer, as reported in Table 1. Since the final response of the SWCNT electrodes depended on the amount of deposited SWCNT, electrodes printed with 12 layers and a SWCNT concentration of 7.5 mg/mL were equivalent to those printed with 18 layers with a concentration of 5 mg/mL. Therefore, a better printability could be obtained with a SWCNT concentration of 5 mg/mL at the expense of the need of printing more layers.

All printed electrodes were electrochemically tested in different solutions in order to determine their capacitance, the electrochemically active area, and the electrochemical reversibility towards redox probes, such as hexacyanoferrate (III/II) and hydroquinone.

Firstly, the capacitive behavior of the electrodes was studied by CV. Figure 3a shows the voltammograms obtained in phosphate-buffered saline (PBS) solution for a Au electrode and SWCNT printed electrodes on a Au. A capacitive behavior can be observed for all electrodes. A significant increase in the capacitive current with the number of printed layers is observed and can be ascribed both to the higher roughness of the electrodes as observed by confocal microscopy (discussed below) as well as the high surface to volume ratio characteristic of nanomaterials. The values of capacitance obtained as the slope capacitive current vs. scan rate were 320, 770 and 1420 µF cm^−2^ for, 6-, 12-(Supplementary Information Figure S4), and 18-layer SWCNT printed electrodes, respectively. The obtained average value of specific capacitance of these SWCNT electrodes was 2.6 F g^−1^ , in accordance with previous reports stating typical values range from 2 to 45 F g^−1^ [37].

Secondly, the electrochemical reversibility of the electrodes was assessed using hexacyanoferrate (III/II) and hydroquinone as redox probes. Figure 3b shows the CVs obtained for a Au and three SWCNT printed electrodes, with 6, 12 and 18 layers, in a solution of 10 mM hexacyanoferrate (III/II). It can be seen that the oxido-reduction of hexacyanoferrate (III/II) was mostly inhibited in as-printed Au electrodes, since no significant anodic or cathodic peaks can be observed. The initial electrochemical response could be greatly improved by cleaning the surface of the Au electrodes, a process known as surface activation [35]. After activating the gold surface, anodic and cathodic peaks could be obtained; however, the observed peak potential difference of 214 mV was rather high. In contrast, well-defined voltammetric peaks were obtained for SWCNTs electrodes. Potential peak differences of 91, 101 and 179 mV were obtained when 6-, 12- and 18-layer electrodes were used, indicating that electron transfer reactions presented a greater degree of electrochemical reversibility for a 6-layer SWCNT electrode. As can be seen, peak current increases with the amount of SWCNT deposited on the electrode, since the peak current peaks for a 18-layer electrode was more than twice as much as the 6-layer SWCNT one.

This considerable increase in peak current observed with the increasing amount of SWCNT can be attributed to an increase in the electroactive area of the electrode. The electroactive area *A* could be determined by recording CVs at different scan rates (Supplementary Information, Figure S5a,b). As expected, the current peak followed a linear dependence (Supplementary Information Figure S5c) on the square root of the scan rate as expressed by the classic Randles-Sevcik Equation (1) [38]:

(1) $$i_{p}=0.4463\;n^{3/2}F^{3/2}\;AC\;{\left(\frac{D}{RT}\right)}^{1/2}v^{1/2}$$

where *i_p_* is the current peak, *n* the number of electrons transferred in the redox event, *F* the Faraday constant, *D* the diffusion coefficient, *C* the concentration of the analyte, *ν* the scan rate, *R* the gas constant and *T* the temperature. Electroactive areas of 1.8, 2.6 and 4.1 mm^2^ were determined for 6-, 12- and 18-layer SWCNT-7.5 printed electrodes. Therefore, SWCNT presented an electroactive area higher than the geometrical area.

This result might be connected to the high roughness of the electrode surface [39]. In order to study this hypothesis, SEM, FIB cross-sections and confocal images of Au and SWCNT printed electrodes were acquired to evaluate the thickness and roughness of these electrodes.

Confocal images in Figure 4 show average thickness and roughness (standard deviation) values for 1-layer Au; 6, 12 and 18-layer SWCNT-5 and SWCNT-7.5 printed electrodes, whose values are detailed in Table 2.

Figure 5a shows the SEM image of the surface of a SWCNT printed electrode, in which the fibrous nature of the CNT can be seen. The cross section made by FIB (Figure 5b) shows the compact layer formed by the printing of Au nanoparticle ink (orange shaded), forming a uniform film of 430 nm thickness with a low roughness. On the contrary, SWCNT printed films with a thickness of 1.4 µm (blue shaded) show a rough surface with the presence of voids in the bulk of the stack layers.

Considering that confocal images were taken on dried electrodes, the increased roughness and electroactive area observed for SWCNT electrodes suggest that SWCNT can penetrate in the solution beyond the diffusion layer and were able to collect redox species from a higher volume than that allowed for planar electrodes under semi-infinite diffusion conditions. Thus, the high values obtained for the electroactive area suggests that SWCNT were not confined to the electrode surface and were able to react with redox species beyond the diffusion layer as usual for planar electrodes.

Figure 3c shows the CVs obtained for an Au printed electrode and a 12-layer SWCNT printed electrode in a solution containing 4 mM hydroquinone in a PBS buffer of pH 7.4. For Au electrodes used without further treatment, the oxidation of hydroquinone was mostly inhibited. Since direct reaction on the underlying Au substrate was mostly absent, the electrochemical response can be attributed to SWCNT, whose electrocatalytic properties to the oxidation of hydroquinone have been previously reported [31]. It is worth noting that the addition of silica nanoparticles did not inhibit the oxidation of hydroquinone by SWCNT. As can be seen in Figure 3c, the voltammogram for SWCNT-SNP-HRP electrodes presented a lower peak current for the oxidation of hydroquinone, possibly as a consequence of a lower electroactive area as the silica nanoparticles are dielectric.

In conclusion, this voltammetric analysis shows that SWCNT printed electrodes present a high capacitance, with values typical for this material. With regard to the electrochemical reversibility, the oxidation of hexacyanoferrate (II) and hydroquinone was mostly inhibited in as-printed Au electrodes. In contrast, SWCNT-7.5 presented a good electrochemical response for both redox probes.

Since both 6- and 12-layer SWCNT showed a good electrochemical response, these numbers of layers were maintained during the fabrication of enzyme electrodes with enzymes immobilized onto nanoparticles. SiO_2_ nanoparticles were chosen because they can be easily prepared with a high degree of quality by the Störber sol-gel process and with a size amenable to IJP (Supplementary Information Figure S6a). Besides, the surface of silica nanoparticles can be functionalized to create anchoring points for biomolecules.

The enzyme-immobilized silica nanoparticles SWCNT ink electrodes were tested for the detection of hydrogen peroxide, using hydroquinone as a redox mediator. The reduction of hydrogen peroxide, catalyzed by HRP, is accompanied by the oxidation of hydroquinone, which acts as an electron donor [31].The formed oxidized species can be electrochemically detected either by CV or by chronoamperometry if the electrode potential is set at a convenient value. Cathodic linear polarization curves present increasing current values with increasing hydrogen peroxide concentration, as can be seen in Figure 6a. Figure 6b shows the current transients obtained at a constant applied potential of −0.23 V before and after the addition of hydrogen peroxide. Similar results were obtained printing 6 and 12 layers, albeit slightly higher current values were obtained for 12-layer electrodes.

Figure 6c shows the current-concentration curve obtained for the 6-layer of SWCNT-SNP-HRP (with 5 mg/mL and 5 mg/mL concentration of SWCNT and SNP-HRP, respectively) at a measurement time of 60 s for electrodes printed 1 day and 90 days after the ink preparation. As can be seen, the performance of both enzyme electrodes was similar. The obtained sensitivity was around 57 +/− 3 µA cm^−2^ mM^−1^, a value comparable to the ones obtained using enzyme electrodes prepared by other means, such as oxygen plasma oxidation of carbon-based electrodes followed by enzyme immobilization [31,40]. Additionally, the stability of enzyme electrodes was also evaluated in similar fashion. Enzyme electrodes were prepared and calibration curves were obtained after 1, 30 and 60 days since preparation. As can be seen in Figure 6d, they exhibited a similar sensitivity. The results presented in Figure 6c,d show that both the bio-ink and the printed enzyme electrodes were highly stable. Enzyme electrodes were printed presenting a similar sensitivity (55 +/− 4 µA cm^−2^ mM^−1^) and a higher stability as previous approaches.

It is worth noting that the concentration of SNP-HRP had a significant impact on the response of the biosensor. It was observed that ink formulations prepared using concentrations of SNP-HRP lower than 1 mg/mL were not enough to cover the surface electrode (Supplementary Information Figure S6b). Therefore, these concentrations of SNP-HRP were not further considered. On the other hand, if the concentration was too high, the electrochemical performance of the SWCNT-SNP-HPR electrode was impaired. When electrodes with concentrations of 5 mg/mL or higher of SNP-HRP were used, repeated measurements showed an increasing signal until a stable, reproducible current value was obtained. This result can be interpreted in terms of a certain blockage of the electrochemical activity of SWCNT-SNP-HPR films, which could be reversed after repeated measurements, possibly due to a reconfiguration in the film structure. On the other hand, electrodes printed with SNP-HPR concentrations lower than 5 mg/mL immediately responded to hydrogen peroxide generating a reproducible electrochemical signal as shown in Figure 6c.

This fact can be further understood by studying the morphological deposition and thickness of the material over the electrode. Figure 7a shows the SEM image of the electrode surface modified with SWCNT-SNP-HPR ink with 7.5 mg/mL and 1 mg/mL concentration of SWCNT and SNP-HRP, respectively. It is possible to distinguish the typical structure that the SWCNTs forms and among them the nanoparticles. When the concentration of SNP-HRP was 5 mg/mL or higher, the top view of the electrode (Supplementary Information, Figure S6c) was completely covered with nanoparticles. In Figure 7b,c, cross sections of 6- and 12-layer of SWCNT-SNP-HPR electrodes are shown. The 2-layer Au, highlighted in orange, presented a thickness of approx. 900 nm, while the thickness of SWCNT-SNP-HPR films was approximately 220 nm and 900 nm for 6-layer (7.5 mg/mL of SWCNTs and 1 mg/mL of SNP-HRP) and 12-layer (5 mg/mL of SWCNTs and 5 mg/mL of SNP-HRP) respectively.

To summarize, enzyme electrodes were successfully printed using a bio-ink containing SWCNT and silica nanoparticles bearing immobilized HRP. The number of printed layers greatly influenced the performance of the enzyme electrodes, and the quantity of SWCNT-SNP-HPR deposited had to be optimized in order to obtain a high sensitivity, covering the electrode surface without forming thick films.

## 4. Conclusions

Enzyme electrodes were successfully printed using a bio-ink containing SWCNT and silica nanoparticles bearing immobilized HRP. The enzyme electrodes retained the catalytic activity for at least 3 months. We have shown that HRP enzymes immobilized onto silica nanoparticles can be used in the formulation of ink to print enzyme electrodes. The catalytic activity remains stable for a longer time allowing for a longer shelf life. Amperometric biosensors were fully printed demonstrating the great potential of this type of technology for the design, development and manufacture of disposable biosensors, thanks to flexibility in design, the small amount of material used and the automation of the multilayer printing process. In combination with simple and accessible electronics, this type of digital production approach is a very attractive option for the volume production of highly stable, inkjet printed biosensors.

## Figures and Tables

**Table 1:** Electrodes printed with different combinations of SWCNT and SNP-HRP concentrations and number of layers, including the estimated masses of SWCNT and SNP-HRP deposited on the electrodes.

SWCNT Concentration (mg/mL)	SNP-HRP Concentration (mg/mL)	Number of Layers	SWCNT Mass (µg)	SNP-HRP Mass (µg)
7.5	0	6	1.5	0
		12	3.0	0
		18	4.5	0
5	0	6	1.0	0
		12	2.0	0
		18	3.0	0
7.5	1	6	1.5	0.2
		12	3.0	0.4
5	1	6	1.0	0.2
		12	2.0	0.4
5	5	6	1.0	1.0
		12	2.0	2.0

**Table 2:** Average thickness and roughness of electrodes printed with different combinations of SWCNT concentrations and number of layers and a layer of Au printed.

Printed Electrode	Concentration of SWCNT [mg/mL]	Number of Layers	Thickness (ave) [nm]	Roughness (sd) [nm]
Au + SWCNT	5	6	1100	315
		12	1148	340
		18	1452	342
Au + SWCNT	7.5	6	1118	261
		12	1429	290
		18	2107	384
Au	0	1	230	65

## Data Availability

Data is contained within this article and the Supplementary Information.

## Supplementary Materials

The following are available online at https://www.mdpi.com/article/10.3390/nano11071645/s1, Figure S1: Design of printed sensors, Figure S2: Images of a WE printed with SWCNT-5 ink, Figure S3: Waveform and parameters used for printing, Figure S4: Plot of capacitive current vs. scan rate for Au and SWCNTs printed electrodes, Figure S5: CVs obtained for a printed electrode in solution of 10 mM hexacyanoferrate (III/II) and Randles-Sevcik plots, Figure S6: SEM images of SiO2 nanoparticles and WE printed with SWCNT-SNP-HRP inks.
