# Supplementary material for: Fully Inkjet-Printed Biosensors Fabricated with a Highly Stable Ink Based on Carbon Nanotubes and Enzyme-Functionalized Nanoparticles
Source: Nanomaterials (Basel). 2021 Jun 23;11(7):1645. doi: 10.3390/nano11071645 (PMC8303974; doi:10.3390/nano11071645)
Supplement: Supplementary file 1 [file nanomaterials-11-01645-s001.zip › nanomaterials-1267878-supplementary.pdf]

Supplementary Information

Fully Inkjet-Printed Biosensors Fabricated with a Highly Stable Ink based on Carbon Nanotubes and Enzyme-Functionalized Nanoparticles

Mijal Mass [1], Lionel S. Veiga [1], Octavio Garate [1], Gloria Longinotti [1], Ana Moya [2,†], Eloi Ramón [2], Rosa Villa [2,3], Gabriel Ybarra [1,*] and Gemma Gabriel [2,3,*]

[1] INTI-Micro y Nanotecnologías, Instituto Nacional de Tecnología Industrial (INTI), San Martín, Buenos Aires B1650WAB, Argentina; mmass@inti.gob.ar (M.M.); lveiga@inti.gob.ar (L.S.V.); ogarate@inti.gob.ar (O.G.); glonginotti@inti.gob.ar (G.L.)

[2] Institut de Microelectrònica de Barcelona, IMB-CNM (CSIC), Campus Universitat Autònoma de Barcelona, Cerdanyola del Vallès 08193, Barcelona, Spain; ana.moya@eurecat.org (A.M.); eloi.ramon@imb-cnm.csic.es (E.R.); rosa.villa@imb-cnm.csic.es (R.V.);

[3] CIBER de Bioingeniería, Biomateriales y Nanomedicina (CIBER-BBN), 28029 Madrid, Spain

* Correspondence: gybarra@inti.gob.ar (G.Y.); gemma.gabriel@imb-cnm.csic.es (G.G.)

† Present Addresses: Eurecat, Centre Tecnològic de Catalunya, Functional Printing & Embedded Devices Unit, Mataró 08302, Spain

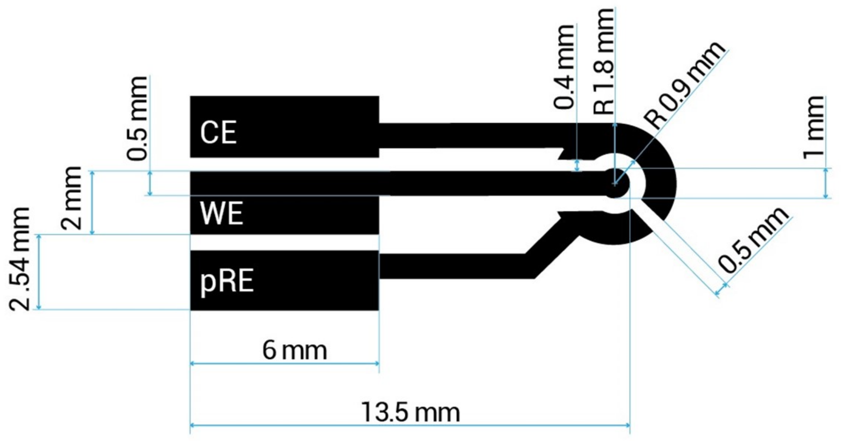

Figure S1. Design of printed working electrode (WE), counter electrode (CE), pseudo-reference electrode (pRE).

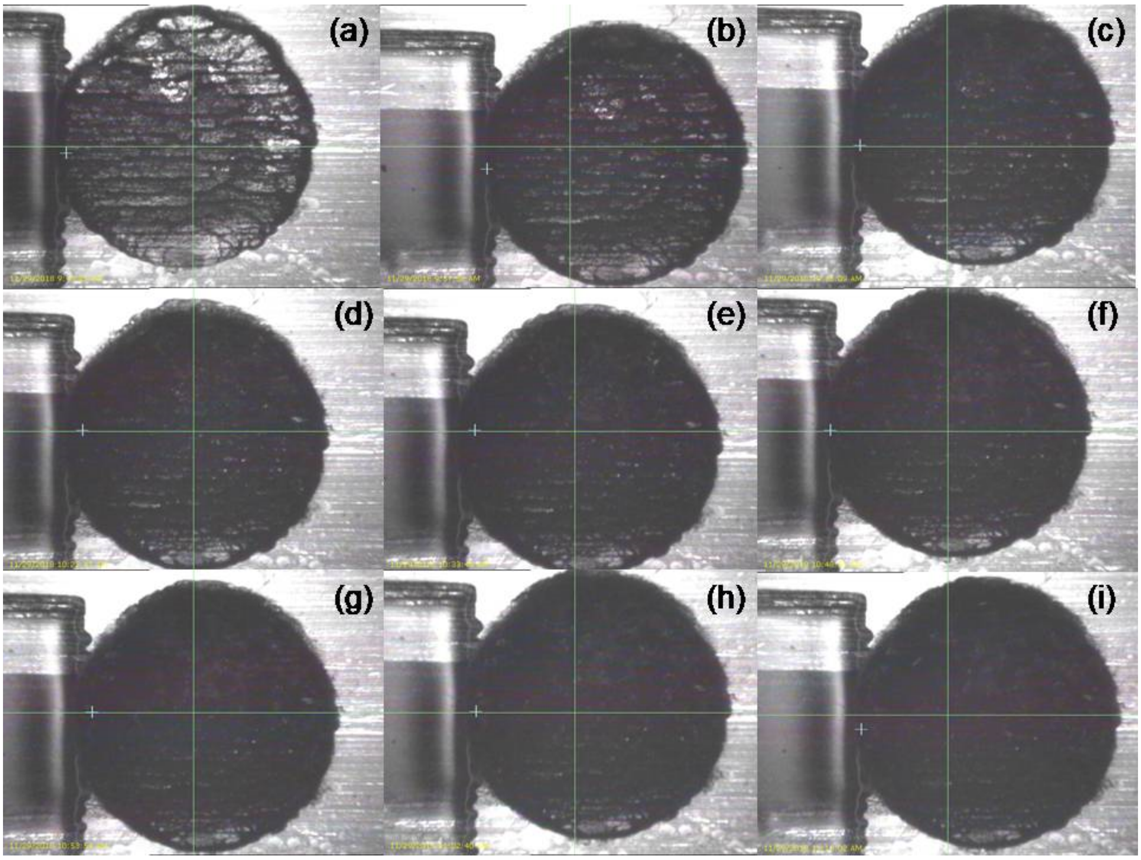

Figure S2. Images of a WE printed with different layers of the SWCNT-5 ink obtained with the camera of the DMP-2831: (**a**) 1-layer; (**b**) 3-layer; (**c**) 6-layer; (**d**) 7-layer; (**e**) 9-layer; (**f**) 12-layer; (**g**) 13-layer; (**h**) 15-layer and (**i**) 18-layer.

(a)

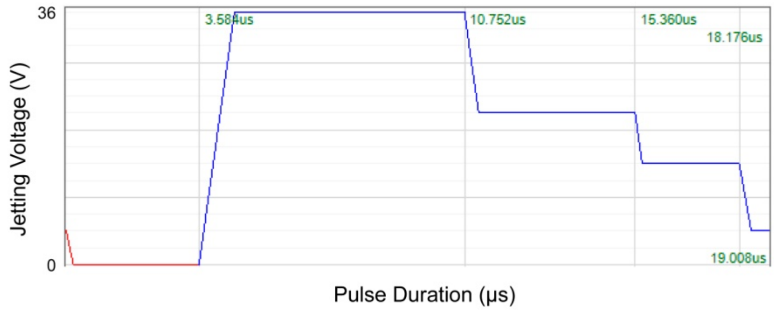

Parameter	Value
Print resolution / Drop spacing	1693 dpi / 15 µm
Maximum jetting frequency	5 KHz
Maximum jetting voltaje	36 V
Maximum number of nozzles	2

(b)

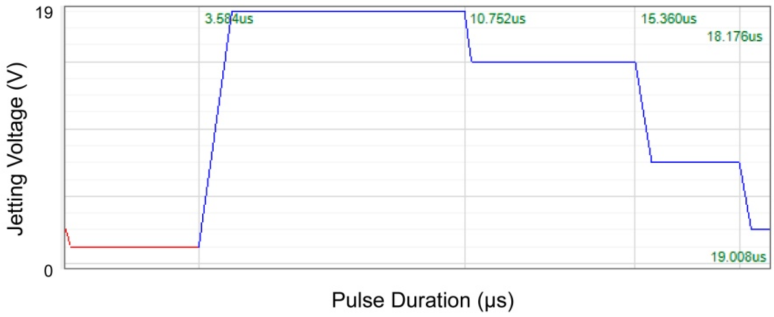

Parameter	Value
Print resolution / Drop spacing	1693 dpi / 15 µm
Maximum jetting frequency	5 KHz
Maximum jetting voltaje	19 V
Maximum number of nozzles	7

Figure S3. Waveform and parameters developed to fine-tune the drop ejection of the inkjet printhead for (**a**) SWCNT-7.5 and (**b**) SWCNT-5.

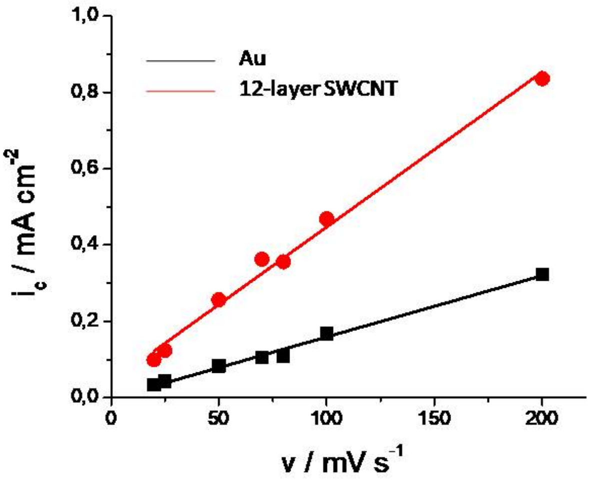

Figure S4. Plot of capacitive current vs. scan rate obtained for a Au printed electrode (black) and a 12-layer of 7.5 mg/ml concentration of SWCNT printed electrode (red).

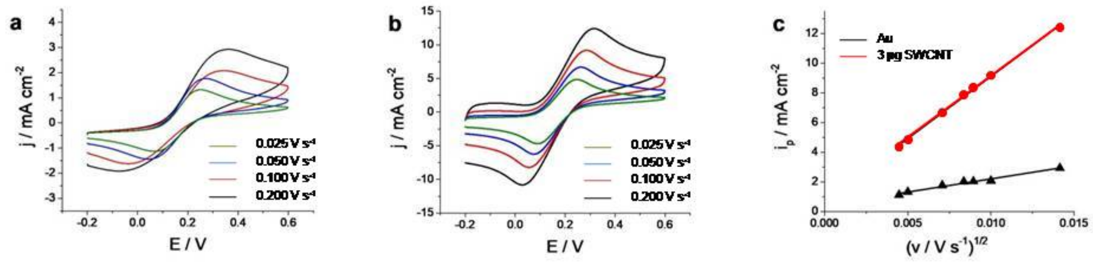

Figure S5. Cyclic voltammograms obtained for a printed electrode in solution of 10 mM hexacyanoferrate (III/II) in a 0.1 M KNO_3 at different scan rates of 0.0250, 0.050, 0.100 and 0.200 V s^{-1}: (**a**) Au; (**b**) with 3 µg of SWCNTs; (**c**) Randles-Sevcik plots.

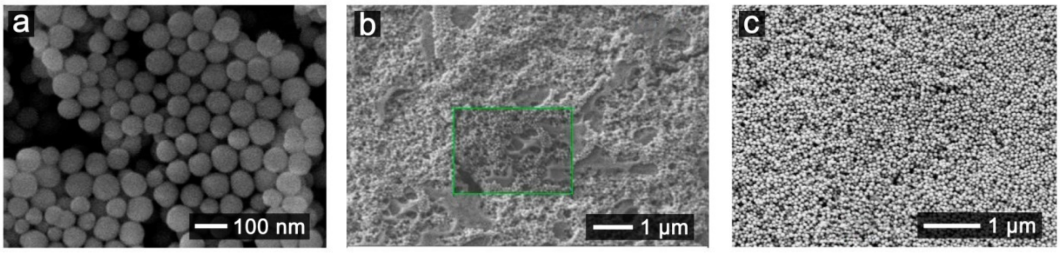

Figure S6. SEM images (**a**) of spherical SiO_2 nanoparticles with an average diameter of 90 nm prepared by a sol-gel process; (**b**) top view of surface WE printed with SWCNT-SNP-HRP ink with concentration of SNP-HRP lower to 1 mg/ml and (c) top view image of surface of 12-layer SWCNT-SNP-HRP (5 mg/ml of SWCNTs and 5 mg/ml of SNP-HRP) printed electrode.
